# Experimental Investigations on the Repeatability of the Fire-Resistance Testing of Electric Vehicle Post-Crash Safety Procedures

**DOI:** 10.3390/s25030688

**Published:** 2025-01-24

**Authors:** Daniel Darnikowski, Magdalena Mieloszyk

**Affiliations:** 1 Maritime Advanced Research Centre, Szczecinska 65, 80-392 Gdansk, Poland; daniel.darnikowski@cto.gda.pl; 2 Institute of Fluid Flow Machinery, Polish Academy of Sciences, Fiszera 14, 80-231 Gdansk, Poland

**Keywords:** electric vehicles (EVs), lithium-ion batteries (LIBs), battery energy storage systems (BESS), thermal runaway, pool fire, safety standards, repeatability, Regulation No. 100

## Abstract

The widespread adoption of electric vehicles (EVs) has elevated the importance of rigorous safety standards, particularly for fire resistance in post-crash scenarios. Existing testing protocols, such as Regulation No. 100, utilize petrol pool fires to simulate real-world fire hazards but lack comprehensive analysis regarding their repeatability and reliability. This study addresses this critical gap by evaluating the variability and consistency of fire-resistance tests performed on multiple battery energy storage systems (BESSs) under standardized conditions. A custom-built measurement system incorporating thermocouples, anemometers, and hygrometers provided high-resolution data on flame dynamics, ambient conditions, and pool fire efficiency. Statistical evaluations following ISO 5725 series guidelines revealed substantial inconsistencies, including unstable exposure temperatures and sensitivity to local turbulence. These findings call into question the robustness of current testing methods, and we propose an alternative approach employing LPG burners for improved precision and repeatability. By identifying significant flaws in existing standards and offering scientifically grounded enhancements, this work contributes a novel perspective to the field of EV safety, advancing global fire-resistance testing protocols.

## 1. Introduction

The advent of electric vehicles (EVs) in recent years has exponentially increased the number of power-train applications for lithium-ion batteries (LIBs) [1]. Lithium-ion batteries represent 90% of the EV battery market, and, in fact, nearly all mass-market electric cars produced today rely on lithium-ion chemistry [2], while other electrochemical energy storage systems are still emerging (e.g., sodium-sulfur, sodium-ion) or are not sufficiently energy dense. Batteries are incorporated into large rechargeable energy storage systems (REESS or RESS) or battery energy storage systems (BESS), which power our vehicles. RESS or BESS are constantly being developed and expanded in size and, by extension, power, and capacity [3] in order to provide a better alternative to internal combustion vehicles (ICE). Regarding the future forecast, EV growth is expected to reach 31.1 million sold units per year in comparison to 2.5 million in 2020 [4,5].

A recent safety report shows the EVs are less likely to cause fires than their ICE counterparts (0.03% for EVs compared to 1.5% for ICE and 3.4% for hybrid vehicles) [6]. This, however, does not take into consideration the severity of the fire, which is subjectively more extreme in EVs and more difficult to extinguish [7,8]. Consequently, this can cause serious damage to the surrounding area and structures [9], as well as a possibly lethal dose of toxic gases [10]. EV fires are predominantly caused by the phenomenon called thermal runaway. It is induced by internal and external factors that lead to a rapid and uncontrollable increase in temperature. Internal factors include manufacturing defects, dendrite formation during charging, and separator failure, which can cause internal short circuits. Overcharging or over-discharging the battery can also destabilize the electrolyte, triggering exothermic reactions [11]. External factors, such as exposure to high ambient temperatures, mechanical impacts, or punctures, can further exacerbate these internal weaknesses [12,13]. Once started, thermal escape can release flammable gases and spread to neighboring cells, significantly amplifying the hazard.

Monitoring thermal runaway in lithium-ion batteries is critical for ensuring safety and preventing catastrophic failures. A variety of sensors are employed to detect early signs of thermal runaway:

- Temperature Sensors: Devices such as thermocouples, resistance temperature detectors (RTDs), and infrared sensors monitor cell temperatures in real time. Advanced methods include embedding fiber Bragg grating (FBG) sensors within battery cells to detect internal temperature variations, providing early warning of potential thermal runaway events [14].
- Gas Sensors: These sensors detect the emission of gases like carbon dioxide and volatile organic compounds (VOCs) during the early stages of thermal runaway. Monitoring gas concentrations within the battery pack enables prompt detection of faults, enhancing safety measures [15].
- Pressure Sensors: These sensors identify swelling or venting events caused by gas generation within the battery cell, which can be indicative of thermal runaway progression [16].

Integrating these sensors into a comprehensive battery management system (BMS) allows for continuous monitoring and the early detection of anomalies, enabling timely intervention to prevent thermal runaway and ensure the safe operation of lithium-ion batteries.

Global reports and articles have shown that LIB fires have largely varying characteristics from petrol-based fires [7]. An outline of the characteristic is provided below along with the recently reported events of such behaviors.

- The battery can re-fire multiple days or weeks after the accident due to slow heat transfer to adjacent modules or cells [17].
- LIB fires produce a range of additional toxic compounds as a result of electrolyte decomposition (e.g., lithium hexafluorophosphate), and these compounds may be released in lethal doses [18], especially in enclosed structures [19,20].
- Battery fires require enormous amounts of water to cool-down and extinguish as the heat capacity of the cells is substantial and their accessibility is heavily impaired [21,22].
- Battery fires are easily spread to nearby structures or vehicles due to their intensity—primarily due to jet flames, explosions, and other flaming components being projected at nearby ignitable objects [1].

With the increasing number of EVs on the road, a rise in fire incidents is expected to happen. The latest reports show that, in China, there has been a 32% year-on-year increase in fire accidents [23]. Additionally, the priority for average EV consumers has been reported to have shifted markedly towards safety concerns, as shown in Table 1 [24]. Despite the fact that driving range still is one of the main disadvantages in most countries, safety concerns with battery technology have almost doubled over two years.

### Test Method Under Investigation

As concerns about the fire safety of lithium-ion batteries (LIBs) increase alongside the growing number of electric vehicles (EVs) on the roads, two key strategies have been simultaneously developed to mitigate these risks: preventing fires through design improvements and rigorous testing protocols, as detailed in previous studies [25], and implementing fire suppression measures once a fire occurs [21]. This article focuses on one of these prevention strategies: the fire-resistance testing procedures outlined in Regulation No. 100—*Uniform provisions concerning the approval of vehicles with regard to specific requirements for the electric power train*, amendments series .02 and .03 (abbreviated as R100.02 and R100.03) [26,27]. The fire-resistance tests described in R100.02 and .03 are mandatory to pass before the LIB is accepted for sale in the EU market. R100.03 contains a test variant in the form of LPG burners instead of pool fire [27]. However, both options are still acceptable for introducing a given product to market. Interestingly, this procedure (only with heat source in form of petrol) is also adopted in other standards, such as ISO 6469-1:2019 [28], ISO 18243:2019 [29], GB/T 31467.3-2015 [30] and GB/T 38031-2020 [31], suggesting that the findings of this study may have broader applicability, including in markets like China [32].

**Figure 1:**
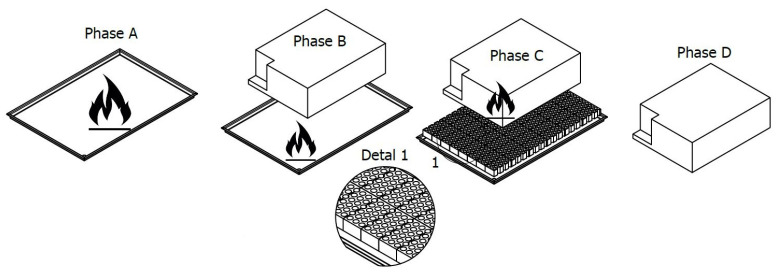
Testing setup and procedure. During Phase A, the fuel burns freely for at least 60 s. In Phase B, the DUT is positioned directly 50 cm above the burning fuel for at least 70 s. In Phase C, a perforated brick screen (shown in Detail 1) is placed between the DUT and the burning fuel for at least 60 s. In the final Phase, D, the DUT is observed for any evidence of an explosion.

The R100.02 and R100.03 fire-resistance testing procedures rely on freely burning pool fires of Positive-ignition fuel, with standardized dimensions and distances between the fire source and the test specimen. The test is divided into four distinct phases, each with a defined duration, as summarized in Table 2 and illustrated schematically in Figure 1. In Phase A, the fuel is ignited and burns freely for 60 s to stabilize the temperature [33]. Phases B and C involve exposing the device under test (DUT) to high temperatures—directly for 70 s in Phase B and indirectly through a perforated screen for 60 s in Phase C (refer to Detail 1 in Figure 1). Finally, in Phase D, the pool fire is extinguished, and the DUT is monitored for any signs of explosion or other hazardous behaviors.

In addition to the prescribed test procedure described above, R100.02 and R100.03 require the following conditions to be satisfied during the test:

1. The test must be carried out in an ambient temperature of at least 20 °C.
2. Wind speed must be limited to 2.5 km/h measured over the fire source.
3. The flame source horizontal dimensions must be larger than the specimen by 20–50 cm.
4. Positive-ignition fuel needs to be used as a fire source.
5. The tested specimen must be set at 0.5 m height from the fire source.

An initial study of the R100.02 fire-resistance procedure revealed significant discrepancies in the heating conditions around the device under test (DUT) [34]. The imposed wind speed limitations were found to be insufficient to mitigate the stochastic nature of the pool fire. Additionally, the optimal steady-state burning mode of the pool fire might not be achieved within the prescribed 60-s pre-burning period, therefore affecting overall exposure conditions. Questions were also raised regarding the pool fire coverage, defined as the ratio of the DUT surface area to the pool fire surface area.

**Figure 2:**
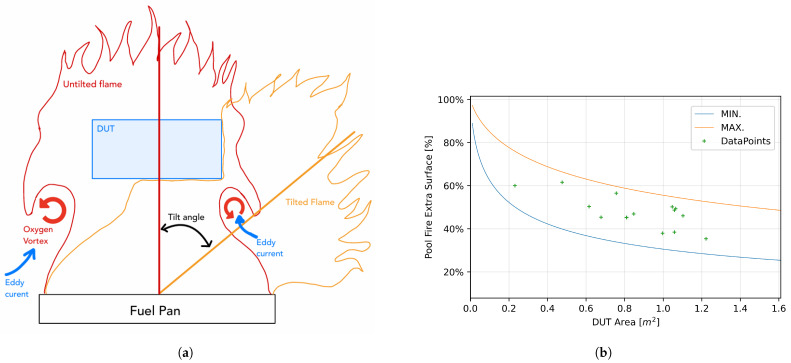
(**a**) Representation of non-tilted flame vortices (red), tilted flame partial engulfment of the DUT (orange), and cold air areas with eddy currents (blue). (**b**) DUT surface area relative to the required pool fire coverage (see Equation (1)). The blue line indicates the minimum excess (DUT size with an additional 20 cm), while the orange line represents the maximum excess (DUT size with an additional 50 cm). Green crosses denote the data points from this study, as detailed in Table 3.

Jiang and Lu [35] demonstrated that crosswind velocities of up to 0.7 m/s can induce a flame tilt angle of 20–40°, significantly reducing the portion of the DUT covered by the fire plume (see Figure 2b). The coverage of a tilted flame was shown to depend on the size and profile of the pool fire, as well as the type of fuel used [36]. At low or zero crosswind conditions, pool fires are prone to buoyancy-induced vortices, which generate lower-temperature regions [37]. These fluctuations affect the exposure temperature at the sides of the DUT, as the cross-sectional area of the pool fire narrows with height—again, dependent on the pool size [38].

Studies indicate that the R100.02 Phase A 60-s pre-burning period may be too short to stabilize exposure temperatures, as the burning model is significantly influenced by the size of the pool fire under study. For large gasoline fires (e.g., greater than 7 m^2^), the optimal burning mode is generally achieved after 50–60 s with a stabilized burning rate ratio [33]. However, lithium-ion battery (LIB) applications of this scale are rare in the power train and day-to-day scenarios [39]. Conversely, Chen and Lu observed that for smaller pool fires, the burning rate ratio evolves [40]. The steady-burning stage for small fires was found to be reached at 210 s [40], compared to the 50–60 s typical for larger fires. Furthermore, a subsequent rise in the burning rate ratio was noted after the steady-burning stage, significantly elevating temperatures.

An important characteristic of pool fires is their area. To quantify fire engulfment of the specimen, a pool fire extra area, $A_{Extra}$, is defined as the proportion of the pool fire area not covered by the tested element. It is expressed as:

(1) $$A_{Extra}=\frac{A_{pool}-A_{DUT}}{A_{pool}}*100\%$$

where $A_{pool}$ is area of the pool fire, $A_{DUT}$ is surface area of the device under test. For instance, a 40% pan excess indicates 60% of the pool fire’s total area is covered by the DUT.

Fire engulfment studies were conducted with an 88.7% excess pool fire area relative to the DUT size [41]. In cases involving large pool fires and much smaller DUTs, convective and radiative heat transfer was found to be evenly distributed around the exposed DUT [42,43]. The R100.02 and R100.03 standards mandate that the pool fire exceed the DUT dimensions by 20–50 cm. As illustrated in Figure 2a, the excess area decreases as the size of the DUT increases.

This study presents a statistical investigation into the repeatability of the R100.02 and R100.03 procedures, analyzing them in terms of exposure conditions, temperature, wind effects, and the sizes of both the DUT and the pool fire. It also offers an evaluation of the reliability of these test methods as safety standards in Europe and China. The purpose of this investigation is to determine the level of repeatability in large-scale pool fire tests involving test objects engulfed in flames and to assess the provisions established in the R100 procedures.

The authors consider this study unique due to the lack of scientific data on fire-resistance tests for large-scale battery packs engulfed in fire and the general absence of research into the repeatability of pool fires as a heating source for fire modeling. Additionally, this paper provides valuable data on large specimens subjected to pool fire testing.

## 2. Experimental Setup and Instrumentation

The experimental site consisted of stationary rectangular pans (tanks without tops) with dimensions provided in Table 3. The pans were filled with 20 L/m^2^ of 95-octane gasoline (E5, as specified in EN 16942 [44]) and 40 L/m^2^ of tap water. The other elements included a movable cart with the specimen, which allowed for transferring the DUT over the pool fire for Phase B, and a cart with a perforated brick screen that could also be moved above the pool fire during Phase C. The exposed surface dimensions of the DUT that were subjected to the tests are listed in Table 3; all samples were of rectangular horizontal projection.

The testing rig was shielded from wind by a 2.5 m-high corrugated steel fence positioned within 30 cm of the tank. At no point did the wind speed exceed the required value of 2.5 km/h. The experimental site was located outdoors, and the ambient conditions—including temperature, humidity, and average wind speed—are listed in Table 3.

The DUT surface areas in the study ranged from 0.23 m^2^ to 1.22 m^2^ while the pool fire sizes varied between 0.58 m^2^ and 2.10 m^2^.

**Figure 3:**
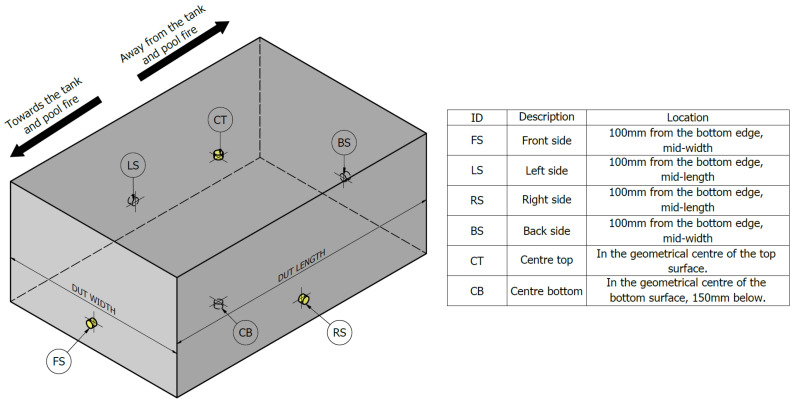
Positioning of the thermocouples around the DUT during the tests.

During the tests, a series of type-K thermocouples were positioned around the DUT to record its surface temperatures. The schematic of the thermocouple placement is shown in Figure 3. The thermocouple measuring tips were mechanically fixed at specified locations on the DUT’s exterior. A total of 60 thermocouples were used, supplemented by two additional thermocouples for ambient and fuel temperature measurements. Each thermocouple was connected to a data logger (Pumpa 0056/2011 with ADAM 4118 RS485 thermocouple modules) with sampling rates ranging from 0.2 Hz to 0.5 Hz. Wind velocity was measured using a Testo 480 hot-wire anemometer. The anemometer probe was positioned at the level of the pan, distanced, and shielded from the fire source to avoid interference from convective heat flows. All measuring equipment was externally calibrated in laboratories compliant with EN ISO/IEC 17025:2018 prerequisites.

The testing rig consisted of rails, two steel carts—one for DUT transport and another for the brick screen—a stationary steel rectangular tank, wind shields made of a 2.5 m high fence, and a 20-foot freight container for DUT storage after the test. The movement of the carts was carried out using a pulley system, operating at a linear speed of 0.4–0.5 m/s. Additionally, a dousing system was enabled to extinguish the burning tank immediately after the DUT was removed from fire exposure.

## 3. Statistical Data Analysis

To quantify the errors and random effects during testing, each test was evaluated using statistical data analysis concepts based on the guidelines and instructions outlined in the ISO 5725 series [45,46,47]. This approach provides numerical insights into the repeatability and precision of the testing procedure and heat source while also contextualizing non-regulated parameters such as ambient temperature, humidity, and flame temperature. Additionally, it allows for comparisons of defined parameters within the testing procedure.

The design of the testing rig is aimed to minimize systematic errors and ensure consistent execution of each test. Random parameters influencing the test results included flame instability, wind gusts, ambient temperature (within allowable limits), humidity, initial petrol temperature, and phase-transition times. These parameters were analyzed for correlations with heating conditions, including temperature and its distribution.

The devices used in the tests were calibrated, and systematic error changes were assumed to have no impact on the system. Measured quantities were treated as random variables following a normal distribution, adhering to the simple acceptance rule [48].

**Figure 4:**
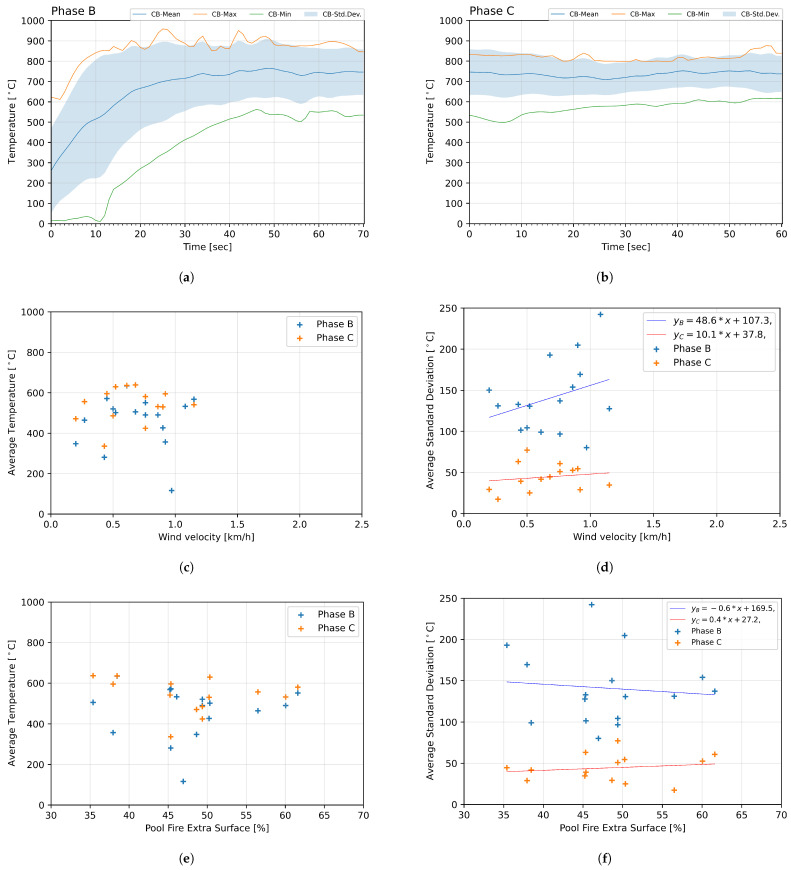
Test results charts. Temperature readings from the thermocouples located at the bottom center of the DUT during Phase B (**a**) and C (**b**). Wind velocity in relation to average standard deviation (**c**) and temperature (**d**) from each test (represented as points). Subsequent figures show pan coverage in relation to the average standard deviation (**e**) and temperature (**f**).

For each thermocouple and test number, the time-dependent mean exposure temperature $\bar{T}$ and standard deviation σ were calculated using the following equations:

(2) $${\bar{T}}_{ij}^{p}\left(t\right)=\frac{1}{n_{ij}^{p}}\sum_{k=1}^{n_{ij}^{p}}T_{ij}^{p}\left(t\right)$$

(3) $$\sigma_{ij}^{p}\left(t\right)=\sqrt{\frac{1}{n_{ij}^{p}-1}\sum_{k=1}^{n_{ij}^{p}}{\left(T_{ijk}^{p}-{\bar{T}}_{ij}^{p}\right)}^{2}}$$

where *i* denotes the thermocouples placement (CT, CB, LS, RS, FS or BS; see Figure 3), *j* is test number (1–16), *i* is the selected phase of the test and *t* represents time. The following data are used in the time–temperature graphs shown in Figure 4a.

Subsequently, the general mean and repeatability standard deviation were estimated for each thermocouple using the following equations:

(4) $$\bar{{\bar{T}}_{i}^{p}}=\frac{\sum_{j=1}^{t}n_{ij}^{p}{\bar{T}}_{ij}^{p}}{\sum_{j=1}^{t}n_{ij}^{p}}$$

(5) $$\sigma_{Ri}^{p}=\frac{\sum_{j=1}^{t}\sigma_{ij}^{p}\;\left(n_{ij}^{p}-1\right)}{\sum_{j=1}^{t}\left(n_{ij}^{p}-1\right)}$$

Currently, there is no available data on the repeatability standard deviation specifically for the R100.03 test. Thus, the calculated $\sigma_{Ri}^{p}$ based on ISO 5275-4 is considered the best estimate [47]. Further checks of precision can only be performed when the repeatability standard deviation of the standard measurement method, $\sigma_{Si}^{p}$, is determined in accordance with ISO 5275-2.

For comparison purposes, the reference standard deviation from the time–temperature curve in ISO 834-1 [49] and the International Code for Application of Fire Test Procedures [50] is used. This standard deviation is based on the maximum allowed tolerance in the standards of ±100 °C, where $\sigma_{Ri}^{p}$ equals 81.6 °C. This value is then used to compute the test statistic *C* and the critical value $C_{crit}$, calculated as follows:

(6) $$C^{p}={\left(\sigma_{Ri}^{p}\right)}^{2}/{\left(\sigma_{Si}^{p}\right)}^{2}$$

(7) $$C_{crit}^{p}=\frac{\chi_{\left(1-\alpha \right)}^{2}\left(v\right)}{v}$$

where $\chi_{1-\alpha }^{2}\left(v\right)$ represents the 1−α quantile of the $\chi^{2}$ distribution with v=j−1 degrees of freedom. For this scenario, α=0.05.

If value $C^{p}$ is larger than $C_{crit}^{p}$, the test method or procedure should be investigated for discrepancies. If $C_{crit}^{p}$ is larger or equal to $C^{p}$, no further investigations are necessary, and the obtained data can be used in subsequent analyses. A summary of the test results is provided in Table 4.

## 4. Results and Discussion

In the following chapter, the authors present and discuss the results of a series of fire-resistance tests. The aim of this analysis is to highlight the crucial aspects of the tests in terms of repeatability and accuracy. Based on the initial study [34], fluctuations and variances were anticipated; however, multiple factors were found to influence the final results.

The analysis focused on Phase B (direct exposure of the DUT to fire) and Phase C (indirect exposure of the DUT to fire), with Phase A included only as a reference for fire stability. Phase D, the observation phase, was intentionally omitted as its outcomes were dependent on the DUT design, which varied significantly between samples and was confidential to the manufacturer. Each of Phases A, B, and C lasted the minimum required durations of 60, 70, and 60 s, respectively. Variations in the total phase lengths were attributed to the movement of the DUT and the screen.

### 4.1. Exposure Conditions

Fire exposure occurs during Phases B and C, where the DUT was positioned directly above the tank. Due to wind variations during the test and eddy currents creating vortices around the flame, it was determined that the thermocouples along the centerline (denoted as “CB”) could reliably evaluate the exposure temperature, as their measuring tips remained within the flame for the entire duration. Figure 4 illustrates the mean (${\bar{T}}_{ij}^{p}\left(t\right)$), maximum, and the minimum temperature, along with the standard deviation ($\sigma_{ij}^{p}\left(t\right)$) during Phase B (Figure 4a) and C (Figure 4b).

During Phase B, a significant variation in starting temperature was observed, ranging from 10.0 °C to 958.3 °C. The mean temperature increased until the 30th second of the phase, where it stabilized at approximately 700 °C. The standard deviation peaked at 270.0 °C and averaged 138.7 °C throughout the phase.

Phase C exhibited relatively stable conditions, with a mean temperature of 740.2 °C and an average standard deviation reduced to 28.4 °C. The observed temperature range during Phase C was between 498.2 °C and 875.7 °C.

For thermocouples located below the DUT (denoted as “CB”), the average temperatures during Phases B and C were relatively stable, indicating that the perforated brick screen did not influence the exposure temperature along the centerline. However, temperature sensors positioned around the DUT (denoted as FS, BS, LS, and RS) showed an increase in values from Phase B to Phase C, though this change was inconsistent. Depending on the location, temperature sensors recorded increases ranging from 73.0 °C to 100.4 °C. The rising temperatures from Phase B to Phase C suggest that the steady-state burning of the pool fire was not achieved, and the regulatory 60-s preheating period may be insufficient to establish stable temperatures. This observation aligns with findings from a previous study [34].

One of the reasons for the significant standard deviation observed during Phase B is that the pool fire had not yet stabilized. The R100 procedure requires a minimum preheating period of 60 s, while studies indicate that the stabilization time may be as long as 150 s to reach the optimum burning rate under given ambient conditions [33]. To address this, either a longer preheating period should be implemented, or the heating medium should be reconsidered. Pool fires have been shown to be prone to puffing and geometry changes due to the entrainment process [51,52]. Vortex formation and the creation of lower-temperature regions at the flame edges may explain the fluctuations observed in thermocouple readings around the tested specimen (see Table 4, denoted as RS, LS, RF, and BS). For both phases, the temperatures around the specimen varied, with notable standard deviations recorded. Studies suggest that proper engulfment of the sample in fire requires a substantially larger pool (above a 1:5 sample-to-pool area ratio) [41,53]. During Phase C, the temperature was observed to be more stable along the centerline, while fluctuations remained significant around the specimen. The use of a perforated brick screen for pool fires between 0.58 m^2^ and 2.10 m^2^ did not affect the temperature readings along the flame centerline compared to Phase B.

The substantial variation in temperature at the start of Phase B can be attributed to two factors: the linear cart speed with the DUT and the DUT size. The testing method does not specify a required linear speed for the carts, which can lead to prolonged exposure of the tested device, as the Phase B timer starts only when the DUT is aligned with the tank’s centerline. Without regulation of cart speed, the exposure period during Phase B can be extended by as much as 29% at an average cart speed of 0.1 m/s. Another influencing factor is the length of the DUT, specifically the dimension parallel to the movement path. Longer specimens remain in the fire significantly longer before reaching the centerline—up to a 68% difference in exposure time can occur between the shortest and longest specimens tested.

### 4.2. Influence of the Wind Speed and Pan-Coverage Ratio

The average wind velocities recorded during the tests ranged from 0.2 km/h to 1.1 km/h, with intermediate values remaining below the regulatory threshold of 2.5 km/h (Section 1). Correlation graphs were generated to analyze the potential influence of wind on the flame temperature around the battery. Average temperature and standard deviation values, calculated from the thermocouples denoted as FS, BS, LS, and RS, were compared with mean wind velocity for Phases B and C, as presented in Figure 4.

Wind velocity was found not to influence the average temperature values on the sides of the tested specimen. However, a correlation was observed between temperature standard deviation and wind velocity, though with a low coefficient of determination. The distribution, shown in Figure 4c, exhibited significant homogeneity. Consequently, the dataset was deemed too sparse to produce a reliable regression model, or the observed relationship may not exist. The average standard deviation was found to be lower during Phase C than Phase B, as shown in Figure 4d.

The test results align with initial findings [34], indicating that hydrocarbon pool fires are prone to fluctuations caused by self-induced air vortices. At wind velocities below 1.1 km/h, temperature results showed no directional bias towards any side, even though the flame tilted periodically during the tests.

The pan-coverage ratio ranged between 38% and 65%. The pan size was determined according to the rules specified in R100.03 [27], with dimensions exceeding the specimen size by 200 to 500 mm.

As with wind speed, correlation graphs were created to investigate the influence of the pan-coverage ratio on exposure conditions, represented by the average temperature during the test phases and the average temperature deviation. The calculations included thermocouples denoted as FS, BS, LS, and RS, which were located on all four sides of the DUT. The graphs are shown in Figure 4.

No correlation was found between the pan-coverage ratio and average temperature during Phases B and C, as shown in Figure 4e. The results demonstrate that an increase in pan-coverage ratio does not influence the temperatures around the DUT. Similarly, the average standard deviation of the temperature (Figure 4f) was not dependent on pan-coverage values. Phase C values for average standard deviation were consistently lower than those for Phase B, mirroring the results observed for wind speed.

### 4.3. Ambient Conditions and Fuel Temperature

Tests were conducted in a range of ambient temperatures above the minimum required 0 °C, with an average of 14.9 °C. Ambient temperature was one of the variables measured to understand its influence on fire resistance and repeatability of conditions. Ambient humidity levels were recorded during each test, ranging from 14.3% to 94.5%. Initial fuel temperature ranged between 3.2 and 27.1 °C. These data were included in the statistical analysis to assess any correlations between humidity and the variability in temperature exposure around the device under test (DUT). However, no statistically significant correlations to exposure temperature and its distribution were found either for the initial temperature of the fuel or for ambient temperature or humidity.

The Pearson correlation coefficients in Phase B for ambient temperature, humidity, and initial fuel temperature are, respectively, −0.29 (*p*-value of 0.273), 0.43 (*p*-value of 0.093), and −0.17 (*p*-value of 0.532), while in Phase C are −0.17 (*p*-value of 0.569), 0.13 (*p*-value of 0.668), −0.08 (*p*-value of 0.793). None of the *p*-values indicate that the correlations are statistically significant.

### 4.4. Discussion on the Test Method

Since the R100 fire-resistance testing procedure is used in the European Union’s homologation process and sets standards for lithium-ion batteries (LIBs) in electric vehicles, the prescribed method should produce repeatable conditions, provided all provisions are met [54]. This testing procedure has also been adopted in other standards, such as ISO 6469-1:2019 [28], ISO 18243:2019 [29], GB/T31467.3-2015 [30], and GB/T38031-2020 [31]. Consequently, similar issues with repeatability may arise globally. Several studies have already highlighted the stochastic nature of hydrocarbon pool fires [32,55,56]. Although a new testing method based on LPG burners was proposed in 2020 [13,32,57], the hydrocarbon curve-based method remains in use for official homologation of battery storage systems [27,58].

According to Table 4 and ISO 5275-4 [47], the Phase B $C^{B}/C_{crit}^{B}$ ratio exceeds 1 for all thermocouple locations except the front side, indicating that the procedure should be reviewed for inconsistencies. All provisions and requirements specified in the standard method (see Section 1) were met, and additional measures were implemented to further limit exposure condition variances. Despite these efforts, large temperature standard deviations were observed throughout Phase B, averaging 132.7 °C. Due to the inherent characteristics of hydrocarbon pool fires, such deviations may be unavoidable. Furthermore, temperature differences between the centerline and the sides ranged from 160.1 °C to 324.9 °C, attributable to the burning dynamics of hydrocarbon pool fires [37,38]. Significant improvements may be difficult to achieve without substituting the heat source. Extending Phase A to at least 150 s [59] could help establish steady-state burning conditions.

LPG burners have been proposed as a replacement for hydrocarbon pool fires in the new GTR No.20 [56], and a custom-made apparatus developed by the Korea Automobile Testing & Research Institute (KATRI) has been suggested as an alternative [57]. However, its practicality remains a topic of debate, particularly regarding the allowed temperature tolerance, which ranges from 800 °C to 1100 °C. An alternative solution involving fire-resistance furnaces has also been proposed [13]. In Europe alone, hundreds of testing facilities are equipped with fire-resistance apparatuses that could potentially be adapted for testing battery energy storage systems (BESS) [60]. Such furnaces typically provide better time–temperature control and can create uniform conditions around the tested elements [32]. Regardless of the selected heat source, a controlled time–temperature curve should be established. Minimum and maximum temperature values should be defined for each test phase to validate the results. For example, a logarithmic heating curve with tolerances of ±100 °C and the area under the curve as a measure of test severity could be implemented.

Phase C exposure conditions showed positive $C^{B}/C_{crit}^{B}$ ratio values in terms of repeatability assessment, as presented in Table 4. This may be attributed to factors such as the influence of the perforated brick screen, which creates a nozzle effect leading to a more stable hydrocarbon pool fire, or the longer burning time, indicating that hydrocarbon pool fires between 0.58 and 2.10 m^2^ require more than 60 s to stabilize. Nevertheless, a similarity in average temperature along the centerline was observed, while the sides exhibited substantial differences, with temperatures ranging between 153.7 and 297.7 °C lower. Since the fire-resistance test for a given DUT is performed only once, a strong bias may develop toward one side. Standardized testing procedures should ensure relatively uniform testing conditions with minimal bias, as real-life pool fires can occur at random locations. The R100 fire-resistance testing procedure thus produces specific conditions that apply to only a narrow range of accident scenarios. Alternative testing procedures, as mentioned in the previous paragraph, could provide more systematic and uniform exposure results around the DUT.

Wind velocity was found not to influence average temperature during Phases B or C. However, an increasing trend in standard deviation with rising wind velocity was observed, as shown in Figure 4d, particularly during Phase B. This indicates the vulnerability of hydrocarbon pool fires to low wind velocity changes, which aligns with observations made during the tests. No tilt bias toward any specific side was detected. Since the mean wind velocity values were relatively low (below 1.1 km/h) compared to other studied conditions [61], it can be assumed that wind velocity does not significantly contribute to the high variance observed during the tests.

The pool fire extra surface was found to be exogenous to both average temperature and standard deviation. For Phase C, the mean standard deviation was significantly lower than in Phase B and remained non-dependent on the pool fire extra surface. As the range of pool fire extra surface is strictly prescribed in the testing method (refer to Section 1), no data are available for DUTs significantly smaller than the pool fire. While increasing the pool fire area may reduce temperature variance around the DUT to some extent, practical challenges arise in designing a reliable testing rig with larger hydrocarbon pool fires, especially when moving parts are involved. These limitations further support the previously proposed transition to LPG burners as a more practical and stable heat source.

The insights gained from this study have valuable implications for computational modeling and simulation of fire-resistance scenarios. The variability observed in the experimental results can serve as critical input parameters for probabilistic models, such as Monte Carlo simulations, to evaluate the range of potential outcomes under similar testing conditions [62]. By incorporating the stochastic elements of hydrocarbon pool fires, such as temperature deviations and flame behavior, these models can better predict real-world performance and inform the design of battery energy storage systems (BESS).

Monte Carlo simulations, in particular, could be used to assess the likelihood of achieving specific temperature thresholds across various DUT configurations. This approach would enable researchers to quantify the influence of key variables, such as wind speed, pan coverage, and preheating time, on test repeatability and safety outcomes. Additionally, integrating these findings into finite element analysis (FEA), computational fluid dynamics (CFD), or large eddy simulation (LES) models could provide a more comprehensive understanding of heat transfer mechanisms and structural integrity under fire conditions [63]. Such simulations could help refine future testing protocols and guide the development of more robust fire safety standards [64].

## 5. Limitations

The pool fire setup introduced inherent inconsistencies in flame behavior due to environmental factors such as wind speed and variations in pan-to-DUT ratios. Despite shielding efforts, flame behavior remained stochastic. The regulatory 60-s preheating phase was found to be insufficient for achieving steady-state burning conditions, particularly for smaller pool fires. This insufficiency likely contributed to significant temperature deviations during Phase B. Additionally, unregulated parameters such as the cart’s linear speed and DUT dimensions impacted exposure durations, introducing further variability into the test results.

Variability in thermocouple placement and sensitivity may have influenced the accuracy and precision of the recorded temperature distributions. While efforts were made to minimize measurement errors, the lack of redundancy in instrumentation remains a limitation. Furthermore, the dataset focused primarily on specific LIB designs and configurations, narrowing the study’s scope and limiting its applicability to the broader range of battery technologies in commercial use.

Although the results are valuable, they are not universally generalizable due to the limited range of battery sizes, chemistries, and configurations tested. Real-world conditions often involve additional complexities that are not accounted for in controlled experimental setups.

## 6. Conclusions

The study identifies significant inconsistencies in current fire-resistance testing methods for lithium-ion batteries (LIBs), particularly in the context of Regulation No. 100. These include flame instability, uneven temperature distributions, and sensitivity to environmental factors such as wind and humidity. The findings reveal that the prescribed preheating time of 60 s is insufficient for stabilizing hydrocarbon pool fires, especially for small-scale setups, leading to substantial variability in test outcomes. Furthermore, the testing rig design required by the standard, including cart movement speed and pool-to-specimen ratio, exacerbates these inconsistencies, compromising the repeatability and reliability of fire-resistance assessments.

The results underscore several shortcomings in the current R100 testing procedures, particularly regarding the inherent instability of hydrocarbon pool fires. Variability in factors such as flame tilt, temperature distribution, and wind conditions significantly affect the repeatability of tests, raising concerns about the reliability of the current methods. Additionally, the study highlights that the preheating times specified may be insufficient for achieving stable fire conditions, leading to inconsistent exposure for devices under test (DUTs). These issues suggest that while the current procedures establish a baseline for LIB fire resistance, they may fail to fully capture the complexities of real-life fire scenarios, thereby limiting their effectiveness in ensuring the safety of electric vehicles (EVs).

To address these challenges, the study proposes alternative testing methods to improve control and precision. The use of LPG burners or fire-resistance furnaces is recommended to achieve more consistent temperature profiles and exposure conditions. Modifications to testing rigs, such as regulating cart speeds and extending preheating durations, are also suggested to enhance test reliability. Additionally, simulation models like Monte Carlo simulations and finite element analysis (FEA) are proposed to better predict fire-resistance performance under varied conditions, offering valuable insights for battery system design.

For practical applications, designers and mechanical engineers should prioritize integrating advanced safety features into battery energy storage systems (BESS). These features include distributed temperature sensors and modular battery structures to localize thermal runaway and mitigate fire risks. Furthermore, adopting fire-resistant materials and protective housings capable of withstanding the temperatures outlined in this study can significantly enhance system resilience. Advocating for globally standardized testing protocols and adopting these innovations will not only improve compliance with safety regulations but also ensure the development of safer and more reliable electric vehicle battery systems.

## Figures and Tables

**Table 1:** Consumer priorities for EV adoption—2018 and 2020 [24].

What Is Your Greatest Concern Regarding All Battery-Powered Electric Vehicles?												
	**France**		**Germany**		**Italy**		**UK**		**China**		**US**	
	**2018**	**2020**	**2018**	**2020**	**2018**	**2022**	**2018**	**2022**	**2018**	**2022**	**2018**	**2022**
Driving range	31%	28%	35%	33%	4%	27%	26%	22%	25%	22%	24%	25%
Cost/premium price	32%	22%	22%	15%	19%	13%	24%	16%	9%	12%	26%	18%
Lack of EV infrastructure	16%	22%	20%	25%	44%	32%	22%	33%	18%	20%	22%	29%
Safety concerns with technology	4%	11%	5%	10%	7%	10%	6%	12%	22%	31%	8%	13%

**Table 2:** Fire-resistance testing procedure outline [26,27].

Phase	Description	Duration	Pass Criterion
A	Preheating	60 s	No explosion i.e., sudden release of energy sufficient to cause pressure waves and/or projectiles that may cause structural and/or physical damage to the surrounding of the Tested-Device.
B	Direct exposure to flame	70 s	
C	Indirect exposure to flame (through perforated screen)	60 s	
D	End of the test	Conditional	

**Table 3:** Information on the dataset: test method, ambient temperature, humidity, average wind velocity, initial fuel temperature, DUT length and width, pool fire length and width, and DUT coverage.

Test No	Ambient Temp., °C	Ambient Humidity, %	Wind Velocity, km/h	Fuel Initial Temp., °C	DUT Length, mm	DUT Width, mm	DUT Surface, m^2^	Pool Length, mm	Pool Width, mm	Pool Surface, m^2^	Pool Extra Area %
1	18.3	68.1	1.0	18.9	1056	802	0.85	1456	1096	1.60	46.9
2	31.2	37.1	1.1	23.4	1365	808	1.10	1685	1214	2.05	46.1
3	11.2	77.5	0.7	13.3	1688	724	1.22	1950	970	1.89	35.4
4	8.4	66.1	0.4	15.1	1051	770	0.81	1480	1000	1.48	45.3
5	16.8	42.1	0.2	24.8	1323	800	1.06	1710	1205	2.06	48.6
6	17.3	28.2	0.9	16.4	1320	793	1.05	1710	1230	2.10	50.2
7	17.3	28.2	0.9	11.0	1208	825	1.00	1460	1100	1.61	37.9
8	27.6	20.8	0.9	26.4	534	433	0.23	760	760	0.58	60.0
9	31.6	25.9	0.5	27.1	830	742	0.62	1240	1000	1.24	50.3
10	3.9	90.0	0.5	3.9	953	711	0.68	1240	1000	1.24	45.4
11	4.5	94.5	0.3	4.5	1364	555	0.76	1740	1000	1.74	56.5
12	5.0	73.6	0.5	5.0	1331	800	1.06	1710	1230	2.10	49.4
13	3.2	69.4	0.8	3.2	1331	800	1.06	1710	1230	2.10	49.4
14	9.4	14.3	0.8	8.4	858	555	0.48	1240	1000	1.24	61.6
15	10.1	21.7	1.2	8.4	1054	769	0.81	1480	1000	1.48	45.2
16	22.8	46.9	0.6	25.5	1323	800	1.06	1720	1000	1.72	38.2
** $\bar{X}$ **	14.9	50.3	0.7	14.7	1162	730	0.86	1518	1065	1.64	47.9

**Table 4:** Summary of the exposure temperatures for Phases B and C—average temperature ($\bar{{\bar{T}}_{i}^{p}}$), repeatability standard deviation ($\sigma_{Ri}^{B}$) and $C^{p}/C_{crit}^{p}$ ratio for thermocouples CT, CB, RS, LS, FS, BS.

	*i*	CT	CB	RS	LS	FS	BS	$\bar{X}$
Value								
$\bar{{\bar{T}}_{i}^{B}},$ °C		325.5	667.0	463.2	476.0	342.1	506.9	463.4
$\sigma_{Ri}^{B},$ °C		123.5	138.7	127.5	158.5	84.0	164.1	132.7
$C^{B}/C_{crit}^{B}$		1.4	1.7	1.5	2.2	0.6	2.5	1.6
$\bar{{\bar{T}}_{j}^{C}},\;$ °C		360.6	740.2	536.2	574.1	442.5	586.5	540.0
$\sigma_{Ri}^{C},$ °C		45.7	28.4	43.7	49.9	33.1	47.0	41.3
$C^{C}/C_{crit}^{C}$		0.2	0.1	0.2	0.2	0.1	0.2	0.2

## Data Availability

Data are contained within the article.

## Abbreviations

The following abbreviations are used in this manuscript:

EV	Electric vehicle
REESS, RESS	Rechargeable energy storage system
BESS	Battery energy storage system
ICE	Internal combustion vehicle
LIB	Lithium-ion battery
R100.02	Regulation No. 100—Uniform provisions concerning the approval of vehicles with regard to specific requirements for the electric power train, amendments series .02 [26]
R100.03	Regulation No. 100—Uniform provisions concerning the approval of vehicles with regard to specific requirements for the electric power train, amendments series .03 [27]
DUT	Device under test
RTD	Resistance temperature detectors
VOC	Volatile organic compounds
LPG	Liquefied petroleum gas
$A_{Extra}$	Fire engulfment of the specimen area
$A_{pool}$	Area of the pool fire
$A_{DUT}$	Area of the surface area
$\bar{X}$	Mean value
$\bar{T}$	Temperature mean value
σ	Standard deviation value
*i*	Thermocouples placement (CT, CB, LS, RS, FS or BS; see Figure 3)
*j*	Test number (1–16) (see Table 3)
*t*	Time of the test
*p*	Phase of the test (A, B or C)
$\sigma_{Ri}^{p}$	Repeatability standard deviation of given thermocouple *i* in given phase *p*
*C*	Test statistic (according to ISO 5725-2 series)
$C_{crit}$	Critical value for statistical tests (according to ISO 5725-2 series)
1−α	Quantile of the $\chi^{2}$ distribution
*v*	Degrees of freedom
*j*	Number of tests
